# A Comparative Analysis of Peripheral Dose Measurement Between Apex and Agility Collimators in Linear Accelerators

**DOI:** 10.31557/APJCP.2026.27.1.287

**Published:** 2026-01-21

**Authors:** Minnu N Tomy, Shambhavi C, Sarath S Nair, Jyothi Nagesh, Shirley Lewis, Krishna Sharan, Sajeesh S Nair, Sumeer Hussain, Vani Lekshmi R

**Affiliations:** 1 *Medical Radiation Physics Programme, Manipal College of Health Professions Manipal, Manipal Academy of Higher Education, Manipal-576104, Karnataka, India. *; 2 *Department of Radiation Oncology, Kasturba Medical College, Manipal Academy of Higher Education, Manipal 576104, Karnataka, India.*; 3 *Department of Radiotherapy, K S Hegde Cancer hospital NITTE university Deralakatte Mangalore India.*; 4 *Department of Radiotherapy, Dr K M Cherian Institute of Medical Sciences, Kallissery P.O, Chengannur, Kerala, India. *; 5 *Department of Radiation oncology, AL Moosa Specialist Hospital AL Ahsa, Saudi Arabia. *; 6 *Department of Health Technology and Informatics Prasanna School of Public Health Manipal, Manipal Academy of Higher Education, Manipal-576104, Karnataka, India. *

**Keywords:** Peripheral Dose, Linear Accelerator, Multi-Leaf Collimator, Apex, Agility

## Abstract

**Objective:**

The aim of this study is to compare and evaluate peripheral dose (PD) distributions from Apex and Agility multileaf collimators (MLCs) in a linear accelerator at varying beam energies, field sizes, and depths for the optimization of radiotherapy safety and precision.

**Methods:**

PD values were determined with Semiflex and PinPoint ionization chambers at depths of 2, 4, 8, and 10 cm in a water phantom. Doses were measured at distances of 1 - 5 cm from the field edge, for photon beams of 6 FF, 6 FFF, 10 FF, and 15 FF MV, with field sizes of 5 × 5 cm² and 10 × 10 cm². The measurements were normalized to the central-axis dose.

**Results:**

The PD varied with depth and beam energy but decreased with increasing distance from the edge of the field. The PinPoint chamber consistently reported lower doses near the field edge (maximum of 63.6% and 66.4%) in Apex and Agility compared to the Semiflex chamber (89.7% and 87.9%) respectively. Agility MLC tended to deliver higher peripheral doses than apex, reaching as high as 87.9% at 10 cm depth and 1 cm distance whereas with Agility, the value was 65.9% under the same conditions.

**Conclusion:**

The PD depends on the design of the MLC, photon energy, depth and beam size. The consistent reduction in peripheral dose with Apex and FFF beams is an encouraging finding that supports their continued use in advanced radiotherapy techniques and clinical decision-making.

## Introduction

Cancer, a prevalent global disease in which abnormal cells divide uncontrollably, can be treated through various treatment modalities, such as chemotherapy, immunotherapy, surgery and radiotherapy. In radiation therapy, high-energy beams are utilized precisely to target tumors, along with minimal damage to surrounding healthy tissues [1].

In a radiation beam, the central axis experiences the highest dose, which falls off towards the field edge; for flattened beams the reduction follows a sigmoidal pattern rather than a strict exponential to approximately 50% at the border of the field [2]. The penumbra effect at the field boundary plays an essential role in treatment planning and outcomes for cancer patients by influencing dose distribution and conformity [3, 4]. Additional margins are crucial to accommodate the penumbra, ensuring that the dose is sufficient to reach the target area. However, the Peripheral Dose (PD) presents risks of undesirable side effects in areas not targeted, particularly for long-term cancer survivors [4]. Pediatric patients exhibit heightened radiosensitivity and a longer lifespan, making them susceptible to secondary malignancies, developmental abnormalities, and chronic organ damage even from low levels of out-of-field radiation. Similarly, in adults, it is crucial to limit peripheral dose when treatment areas are in proximity to radiosensitive organs like the eyes, thyroid, heart, gonads, or breasts, as even minor unintended radiation exposure can lead to significant adverse effects. Even a small fraction of the total treatment can lead to injury or secondary cancer. For example, fetal damage can occur at doses as low as 0.05 Gy, with the risk becoming considerable between 0.1 and 0.5 Gy [5-9]. The magnitude and spatial distribution of peripheral dose depend heavily on treatment technique, beam modulation, and collimator design. For this reason, systematic evaluation of collimator performance is essential, especially in high-precision modalities such as IMRT and VMAT, where modulation complexity can increase scatter and leakage [10, 11]. Radiation can escape from three main sources: (1) leakage from the treatment unit (2) scattering from secondary devices such as collimators, wedges, and blocks and (3) Internal scattering from the patient itself. The first two sources are affected by how the treatment unit is set up. This means that modifications to the head design or the addition of beam modifiers can impact on the peripherals dose. The PD has been assessed across various megavoltage photon beams and treatment units [5, 12].

Multi-leaf collimators (MLC) are special tools used in radiation therapy that can adjust to fit the shape of different tumors. Three-dimensional conformal radiotherapy (3D-CRT) helps target tumors more precisely, and Intensity-Modulated Radiotherapy (IMRT), Volumetric Modulated Arc Therapy (VMAT) controls the strength of radiation to protect healthy tissue. These methods require complicated setups with uniquely shaped fields, and the way they are arranged can greatly influence how the dose of radiation is distributed, especially in smaller areas. To ensure accurate treatment, Dosimetric verification of different collimators is essential before clinical implementation. Notably, various linacs vendors offer distinct collimator/MLC designs, highlighting the need for verification [13].

The linear accelerator (LINAC) offers versatile beam delivery, including photon energies of 6, 10, and 15 MV, with an optional Flattening Filter-Free (FFF) capability, alongside electron energies ranging from 4 to 15 MeV. Its expansive 40 × 40 cm² treatment field, defined by sculpted diaphragms, is complemented by advanced MLCs comprising 80 interdigitating pairs. These tungsten MLC, which are part of Elekta’s agility collimator, boast a leaf width of 5 mm and rapid movement speeds of up to 6.5 cm/s [14]. The apex micro-MLC is another type of MLC. Which has 56 pairs of leaves, and each leaf is 2.5 mm thick at the isocenter and has a maximum field size of 12 × 14 cm², apex enables high-dose rate stereotactic treatments via Dynamic Conformal Arc Therapy (DCAT). This advanced configuration of one of the 4 micro-MLC technology (Elekta apex micro-MLC, Brain LAB m3 mMLC, Varian High-Definition MLC (HD120), Siemens (PRIMART / ONCOR with 160-leaf MLC) facilitates the precise and efficient delivery of radiation therapy, leveraging the versatility of VMAT [15]. This crucial component directly influences the distribution of radiation doses, minimizing exposure to healthy tissues and maximizing tumor targeting. By reducing dose fall-off and the penumbra, the MLC apex enables oncologists to deliver radiation with pinpoint accuracy, sparing surrounding tissues and reducing side effects. Moreover, its design significantly impacts treatment planning algorithms, allowing for better optimization of radiation dose distributions. As technology continues to evolve, advancements in MLC design are revolutionizing radiation oncology, enabling more precise conformity of radiation doses to tumor shapes. Ultimately, the MLC apex is a game-changer in the fight against cancer, transforming treatment outcomes and saving lives [8]. This study aimed to compare the peripheral dose characteristics between two MLC systems, apex and agility, with different broad range of beam energies (including FFF modes) and at different depth and chamber with consistent setups and repeated measurements.

## Materials and Methods

### Research Setting and Ethical Clearance

This prospective cross-sectional study was performed at the Department of Radiotherapy Oncology, Kasturba Medical College (KMC), Manipal using Elekta HD versa machine. The study was purely experimental and machine-based and did not involve human or animal subjects. A convenience sampling technique was employed for measurement acquisition. Approval of this study was obtained from the Institutional Ethics Committee of Kasturba Medical College, Manipal (IEC275-2025). Since the research included no human or animal subjects and was carried out with machine-generated data alone, informed consent was not needed.

### Experimental Design

**Figure 1:**
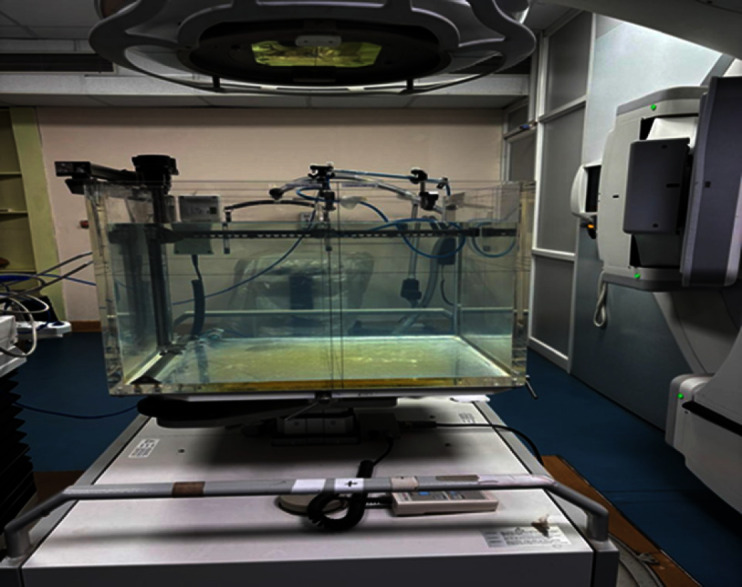
Dosimetric Setup for the Peripheral Dose Measurement Using Semiflex and Pinpoint Chambers Using Water Phantom

For the purposes of ensuring robustness and reproducibility, a block experimental design was adopted that consisted of three replicates for each measurement condition. There were 576 experiments that included combinations of two ionization chamber types semiflex and Pin Point (PTW Freiburg), four measurement depths (2 cm, 4 cm, 8 cm, and 10 cm), four distances from the field edge (1 cm to 5 cm from the field edge), four energies of photon beams (6 MV FFF & 6,10 15 MV FF) and two field sizes (5×5 cm² and 10×10 cm²).² Figure 1). Each measurement point represents an average of three repeated measurements to ensure repeatability; standard deviation was computed to estimate measurement uncertainty.

### Instrumentation and Dosimetry Setup

All the experiments were performed on the same Versa HD LINAC, which can provide high-energy photon beams and is also provided with two MLC systems, namely, apex and agility. These collimators, as elucidated earlier in the introduction for their mechanical and dosimetric benefits, were tested under controlled situations to analyze their effects on the peripheral and depth dose distributions. A water phantom was utilized as the medium measurement because of its uniform density and radiation absorption properties like those of tissue. It provides a reproducible and stable platform for detector positioning at different depths and off-axis distances. Two ionization chambers were used for dose collection: the semiflex chamber, which is generally applied for routine field measurements, and the Pinpoint chamber, which excels in high-resolution performance in areas with steep dose gradients. Prior to measurement, both detectors were calibrated, and the required correction factors were introduced to ensure comparability between the setups.

### Calibration and setup procedure

The linac was calibrated prior to measurement to ensure appropriate photon output, flatness, and symmetry. MLC positioning was verified for apex and agility machines to remove mechanical discrepancies. The solid water phantom was aligned through laser alignment using a fixed SSD of 100 cm. The gantry was positioned at 0°, and beam profiles were set up for 5×5 and 10×10 cm² field sizes. The ionization chambers were set at fixed depths and along the center axis of the beam for accurate measurement, as shown in Figure 1.

### Radiation delivery and data acquisition

**Figure 2:**
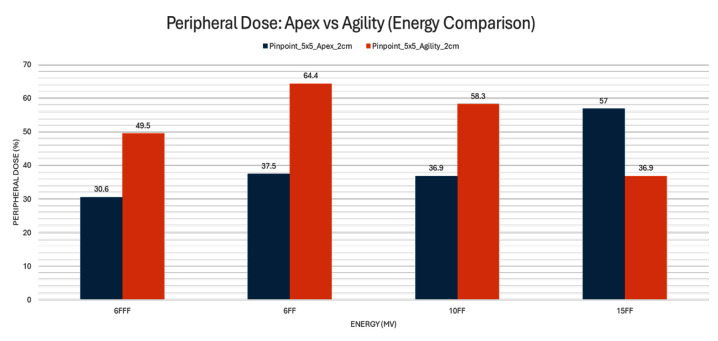
Peripheral Dose Comparison between Apex and Agility Multileaf Collimator Systems Across Different Beam Energies Using a 5×5 cm² Field at 2 cm Depth

Measurements are taken of the photon beams at 6 MV FFF, 6 MV FF, 10 MV FF, and 15 MV FF (Figure 2). The dose was taken at various depths and off-center lateral distances from the edge of the field with Semiflex and Pinpoint chambers for each setting. All the values of the dose were normalized to the central axis at the depth of max, and defined field size of 10x10 cm^2^. The normalized values enabled the computation of Percentage Depth Dose (PDD), which is the relative dose at a particular depth as a percentage of the reference dose.

### Peripheral Dose Evaluation

The PD defined as the dose measured outside the central treatment field, was evaluated as a percentage of the central axis dose. Measurements were performed under varying conditions of depth, distance from the field edge, beam energy, and collimator size. To assess the influence of treatment head design and detector characteristics, the same measurements were repeated using two different MLC systems (agility and apex) and two types of ionization chambers (Semiflex and Pinpoint). These variables were used to estimate the shielding efficiency and scatter properties of the apex and agility collimators. Repeated measures were utilized to assess measurement repeatability and measurement uncertainty.

### Statistical analysis

The collected data were then processed and statistically compared with Jamovi Software version 2.3.28. The Shapiro‒Wilk test was used to determine whether the data were normally distributed. If the data were determined to be normally distributed, independent sample T tests were used to compare group means. When the data were not normally distributed, nonparametric tests were used: the Mann‒Whitney U test was used to compare two independent groups, and the Kruskal‒Walli’s test was used to compare more than two groups. Statistical significance was defined at a p-value < 0.05. All reported p-values were employed to determine if the observed differences in the peripheral dose between varying field sizes, depths, energies, types of chambers, and collimator systems were significant.

## Results

**Figure 3:**
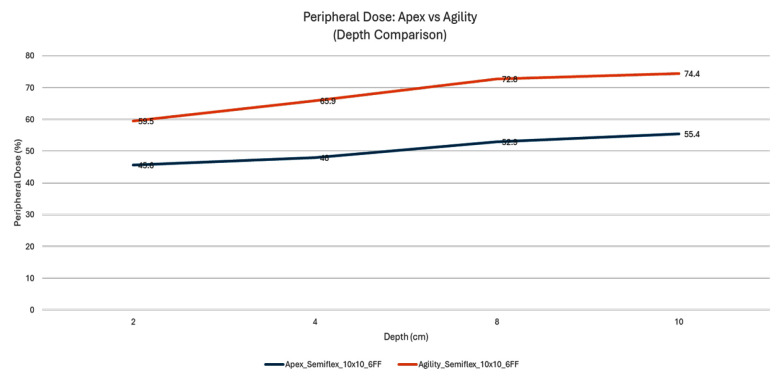
Peripheral Dose Comparison between Apex and Agility Multileaf Collimator Systems at Varying Depths at 10×10 cm² Field with 6 MV Flattened Beam Using Semiflex.

PD exhibited an increase with depth for both the fields, irrespective of beam qualities, MLC configurations, and detectors, which is attributable to dose buildup and electron contamination (Figure 3). Specifically, using the Semiflex chamber, values rose from 45.6% at 2 cm to 65.6% at 10 cm for the 10 × 10 field (Table 2). Correspondingly, Pinpoint measurements with agility showed an increase from 64.4% to 72.0% for the 5 × 5 field over the same depth interval (Table 1).

Beam energy played a significant role in PD for both field sizes. Lower doses were consistently recorded for 6 MV FFF beams (Table 3), whereas higher energies resulted in elevated doses, although the differences between 10 and 15 MV were minimal. For instance, Pinpoint measurements indicated a PD increase from 28.6% with 6 MV FFF to 38.4% with 15 MV FF for the 10 × 10 field. Similarly, using the Semiflex chamber with the 5 × 5 field, values escalated from 39% with 6 MV FFF to 61.7% with 15 MV FF. The MLC system demonstrated a pronounced effect on PD, with agility consistently yielding higher values than apex, particularly near the field periphery. At a depth of 2 cm, using Pinpoint and a 6 MV FF beam, PD increased from 36.9% to 58.5% for the 10 × 10 field and from 37.5% to 64.4% for the 5 × 5 field, representing approximately 22% and 27% increases, respectively.

Detector dependence was observed across all tested conditions. The Semiflex chamber generally measured 5–10% higher than the Pinpoint chamber, with the greatest discrepancies occurring at higher beam energies and greater depths. For example, within the 10 × 10 field, the Semiflex chamber recorded 60% compared to 55% for the Pinpoint chamber, while for the 5 × 5 field, the same configuration yielded 89.7% versus 45.7%. With FFF beam it is noted that the PD reduced for both field sizes. Using the Semiflex chamber, PD in the 10 × 10 field decreased from 45.6% with 6 MV FF to 36.4% with 6 MV FFF. A comparable reduction was noted for the 5 × 5 fields, where Pinpoint measurements decreased from 37.5% to 30.6%.

PD decreased sharply with increasing distance from the field edge for both field sizes. For the 10 × 10 field, the dose diminished from 36.9% at 1 cm to 0.4% at 5 cm, with a similar trend observed for the 5 × 5 field. The apex MLC showed a steeper dose fall-off, whereas the agility MLC maintained comparatively higher doses at greater off-axis distances. Across all measurements, the highest recorded PD was 89.7% with the Semiflex chamber, and the lowest was 0.1% with the Pinpoint chamber.

Statistical analysis via the Kruskal-Wallis and Mann-Whitney U tests revealed significant differences (p < 0.05) in the peripheral dose among the MLC types, energy levels, depths, and chamber configurations. The p values obtained were consistently less than 0.05, confirming the statistical significance of the observed differences. For the comparison between agility and apex MLC, the Mann-Whitney U test yielded a p-value of 0.001, with statistically significant. Additionally, for the comparison between 6FF and 6FFF energies, the Mann-Whitney U test yielded a p-value of 0.006. Pinpoint vs. Semiflex Chambers: The Mann-Whitney U test for these chambers (combined energy) yielded a p-value of 0.067. Since 0.067 > 0.05, the difference in the “Outcome” between pinpoint and semiflex chambers is not statistically significant. This finding indicates that the dose variations are meaningful and not attributed to random chance. Additional analysis confirmed that differences between apex and agility PD were statistically significant across most energies and depths (p<0.05).

## Discussion

**Figure 4:**
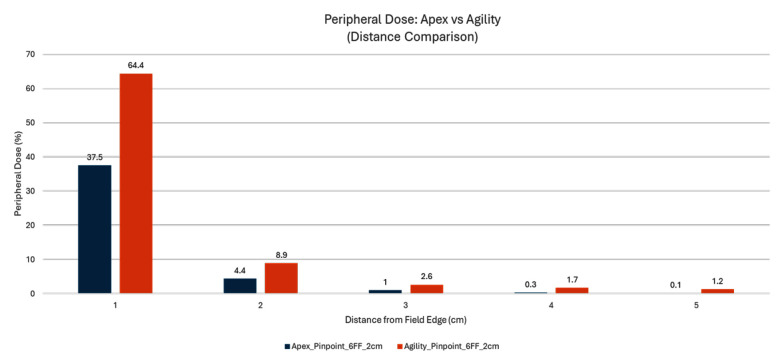
Peripheral Dose Comparison between Apex and Agility Multileaf Collimator Systems at Different Distances from the Field Edge (1–5 cm) using 6 MV flattened beam at 2 cm depth

**Figure 5:**
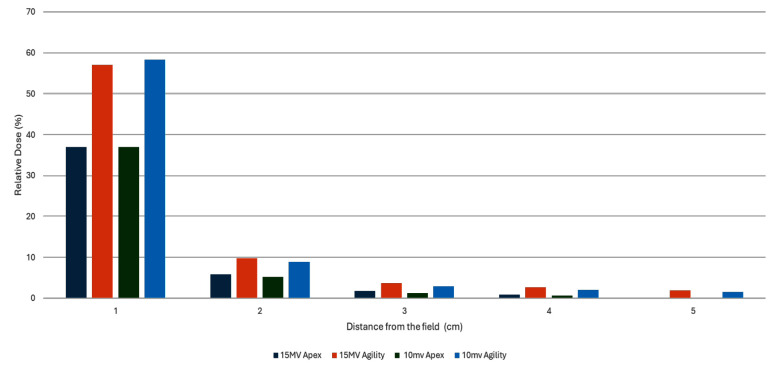
Comparison of Relative Dose Data for Apex and Agility for 15 and 10 MV Photon for 5x5 cm^2^ Field Size

This study explored the PD measurement at different depths, distances from the field periphery, beam energies, and multileaf collimator configurations, employing two ionization chambers. The findings revealed consistent trends, offering valuable insights into the dosimetric properties of modern linear accelerator. Consistent with previous research employing Co-60 and linear accelerator beams, this pattern indicates a rapid reduction in peripheral dose as lateral distance increases [15]. The out-of-field dose decreased rapidly with distance from the field edge for all conditions, confirming that peripheral dose is dominated by scattered radiation that attenuates with increasing distance (Figures 4,5). At 1 cm from the field edge, values were relatively high (10–70% of the central axis dose, depending on depth and energy).

Measurements near the field edge were consistently higher for the agility MLC system compared to apex, particularly at shallow depths and off-axis distances, this suggests that variations in MLC design and leakage characteristics significantly influence out-of-field dose highlighting a need to consider hardware-specific dose behaviors when assessing peripheral doses. At 2 cm depth with 6 MV FF, for example, out-of-field dose 1 cm from the edge was nearly double for agility compared to apex. These differences can be attributed to variations in MLC leaf transmission and interleaf leakage between the two designs [16-18]. These differences are significant when the exposure of organs at risk (OARs) to conformal and intensity-modulated treatments is considered. The agility system, with 160 high-speed, interdigitating leaves and enhanced shielding systems, delivered higher peripheral doses under similar circumstances [19-21].

A clear depth dependence was observed, with deeper measurement depths yielding higher out-of-field doses. The dose outside the primary beam increased with measurement depth, indicating that internal patient scatter contributed more significantly at depths of 8–10 cm than at shallower locations. This finding aligns with previous research using phantoms and patients, which demonstrated that scatter originating from the phantom was the principal factor influencing peripheral dose at greater depths, while head leakage was the main determinant at shallower depths. This effect can be attributed to increased phantom scatter and build-up of scattered photons with depth. For instance, at 6 MV FF, the out-of-field dose at 1 cm increased from approximately 37% at 2 cm depth to over 60% at 10 cm depth as mentioned by Athiyaman H et al [22, 23]. Clinically, this indicates that organs located deeper within the patient may receive higher stray doses than those closer to the surface, even at the same lateral distance from the treatment field. This is because of greater internal scatter and secondary interactions, as already proven in empirical and computational models [24, 25]. The larger field sizes, e.g., 10×10 cm², also demonstrated more peripheral exposure, which is consistent with earlier evidence showing that larger irradiation fields produce more out-of-field radiation owing to greater patient scatter and collimator transmission [26].

Using the Monaco 5.1 treatment planning system It was observed with Monte Carlo calculations for a 6 MV FFF beam and a 10 × 10 cm² field, the surface dose measured 2 cm lateral to the field edge was 1.8 cGy with the agility MLC compared to 1.2 cGy with the apex MLC (for a reference dose of 100 cGy at 100 cm SSD), demonstrating the improved capability of the apex system in reducing peripheral dose. These findings are in line with previous measurements and simulations of MLC systems in comparable configurations [27, 28]. Historical assessments by Stern and Mazonakis further revealed that MLC leakage and collimator scatter are major contributors to peripheral dose distributions in both phantom and clinical models [29, 30].

The PD is clinically significant since it contributes to the integral dose, which can increase the risk of secondary malignancy, particularly for children and long-term survivors [31]. The results here support earlier results that low-level out-of-field doses can be reduced through design changes in MLC and proper treatment planning [32].

Peripheral dose was also affected by beam energy. Higher photon energies generally resulted in increased near-edge doses relative to 6 MV, though these distinctions lessened with greater distances. This observation aligns with the greater scatter contribution and heightened head leakage at elevated energies, with attenuation over distance mitigating the impact of these factors. Notably, FFF beams consistently yielded reduced out-of-field dose compared to flattened beams. The elimination of the flattening filter decreases head scatter, thereby accounting for the observed reduction and substantiating the clinical application of FFF beams for minimizing peripheral dose [33].

The detector type employed had a notable impact on the measured dose values. Specifically, the Pinpoint chamber consistently yielded lower doses at the field periphery compared to the Semiflex chamber, which, conversely, recorded marginally elevated values at greater distances from the field center. This observed divergence can be attributed to the differences in their sensitive volumes; the PinPoint detector, with its smaller volume, offers superior resolution for steep dose gradients, while the larger Semiflex detector integrates more scattered radiation, leading to an overestimation of the dose near the field edge and the capture of additional scatter at larger distances [34]. Consequently, these findings underscore the importance of selecting a detector appropriately. This equipment, when used together with precise beam modeling, enhances peripheral dosimetry and is consistent with commissioning guidelines advised in modern QA standards [35].

The PD in radiotherapy, particularly with agility and apex MLC, is significantly affected by the treatment planning system’s modeling factors, such as leaf transmission, leakage, tongue-and-groove effects, and the leaf tip design. During commissioning, it is crucial to perform direct PD measurements, conduct sweeping gaps and asynchronous tests, and meticulously calibrate transmission and tip offsets to ensure the accuracy of TPS predictions. quality assurance should routinely assess leakage maps and peripheral dose baselines, while also verifying the proper functioning of jaw tracking and leaf-edge positioning mechanisms to mitigate leakage. Recent technological advances in MLC technology, such as layered leaf designs and enhanced TPS modeling, have also been promising in minimizing transmission even further [26, 36]. Coupling these with advanced VMAT and automated IMRT planning algorithms may also further improve dose conformity while sparing healthy tissues [37–41].

From the study it is noted that the apex MLC system produced lower peripheral dose than the agility system under equivalent treatment conditions. Overall, these results reinforce the importance of careful beam energy choice, and MLC system design in minimizing peripheral dose. Clinically, this is crucial for both Dosimetric and clinical significance, reducing unintended exposure to adjacent critical structures and lowering the risk of secondary malignancies, particularly in pediatric or long-survival patients. Additional research should investigate peripheral dose behavior during dynamic deliveries and assess long-term clinical results related to less scatter exposure.

**Table 1:** Measured Relative Peripheral Dose for Apex and Agility for the Field size 5×5 cm^2^ and Different Energies with Pinpoint and Semiflex

			Distance from the field edge									
CHAMBER	DEPTH	ENERGY	APEX					AGILITY				
	(CM)	(MV)	1cm	2cm	3cm	4cm	5cm	1cm	2cm	3cm	4cm	5cm
	2	6 FF	37.50%	4.40%	1.00%	0.30%	0.10%	64.40%	8.90%	2.60%	1.70%	1.20%
		10 FF	36.90%	5.20%	1.30%	0.60%	0.10%	58.30%	8.90%	2.90%	2%	1.50%
		15FF	57%	9.80%	3.70%	2.60%	1.90%	36.90%	5.80%	1.70%	0.80%	0.10%
	4	6 FF	36.10%	7.30%	1.50%	0.60%	0.20%	66.40%	13.50%	3.30%	2.10%	1.50%
		10 FF	40%	7.20%	1.30%	0.40%	0.20%	60.10%	12.50%	3%	1.90%	1.40%
PINPOINT		15FF	58%	12.80%	3.40%	2%	1.40%	39.40%	7.50%	1.20%	0.30%	0.20%
	8	6 FF	46.40%	12.10%	2.60%	1.20%	0.40%	69.40%	21.30%	4.90%	2.90%	2.00%
		10 FF	45.70%	11.70%	2.30%	0.90%	0.30%	63.60%	19.40%	4.30%	2.50%	1.70%
		15FF	62.20%	19.30%	4.40%	2.50%	1.70%	45.70%	11.70%	2.30%	0.90%	0.30%
	10	6 FF	49.30%	14.30%	3.10%	1.50%	0.60%	72.00%	25.70%	5.60%	3.40%	2.40%
		10 FF	48.60%	13.80%	2.80%	1.20%	0.40%	65.60%	22.50%	4.90%	2.80%	1.90%
		15FF	63.60%	22.10%	4.90%	2.70%	1.80%	48.10%	13.70%	2.40%	0.70%	0.30%
	2	6 FF	44.80%	5.70%	1.60%	1%	0.60%	67.30%	9.90%	2.90%	2%	1.50%
		10 FF	61..4%	5.90%	2.20%	1.50%	1.10%	63%	10%	3.30%	2.30%	1.80%
		15FF	61.70%	11%	4.10%	2.90%	2.20%	56.30%	6.70%	2.40%	1.50%	1.10%
	4	6 FF	48%	9.10%	2.20%	1.30%	0.80%	69.10%	14.60%	3.70%	2.30%	1.70%
SEMIFLEX		10 FF	77.90%	11.10%	4.00%	2.50%	1.80%	64.40%	13.90%	3.50%	2.20%	1.60%
		15FF	62.40%	14.40%	3.90%	2.30%	1.70%	66.40%	8.70%	2.80%	1.60%	1.10%
	8	6 FF	52.90%	14.90%	3.60%	2.20%	1.40%	72.80%	23.40%	5.40%	3.30%	2.40%
		10 FF	89.70%	20.10%	6.60%	4.20%	3.10%	67.50%	21.30%	4.90%	2.80%	2.10%
		15FF	67.50%	21.30%	4.90%	2.80%	2.10%	74.20%	10%	3.80%	2.20%	1.60%
	10	6 FF	55.40%	17.60%	1.80%	1.10%	0.90%	74.40%	27.40%	6.20%	3.80%	2.70%
		10 FF	80.50%	12.60%	5.40%	3.60%	2.70%	69.50%	24.80%	5.50%	3.20%	2.30%
		15FF	67.90%	24.60%	5.70%	3.20%	2.30%	87.90%	20.70%	6.50%	3.80%	2.70%

**Table 2:** Measured Relative Peripheral Dose for Apex and Agility for the Field size 10×10 cm^2^ and Different Energies with Pinpoint and Semiflex

			Distance from the field edge									
CHAMBER	DEPTH	ENERGY	APEX					AGILITY				
	(CM)	(MV)	1cm	2cm	3cm	4cm	5cm	1cm	2cm	3cm	4cm	5cm
	2	6 FF	36.90%	6.30%	1.70%	0.90%	0.40%	58.50%	12%	4.20%	3.10%	2.30%
		10 FF	37.30%	7.40%	2.30%	1.10%	0.60%	59.60%	17%	7.50%	3.80%	2.90%
		15FF	38.40%	8.50%	2.90%	1.70%	1%	58%	16%	7.10%	3.40%	2.70%
	4	6 FF	44%	10.40%	2.60%	1.40%	0.80%	61.90%	18%	5.20%	3.70%	2.70%
		10 FF	43.30%	10%	12%	1%	0.40%	61.80%	20.60%	7.80%	4.50%	4%
PINPOINT		15FF	44%	10.50%	2.10%	0.80%	0.30%	60%	19.20%	6.90%	4.20%	3.40%
	8	6 FF	55.80%	17.70%	4.80%	2.80%	1.70%	70.20%	29.70%	7.80%	5.30%	3.90%
		10 FF	55%	17%	4.10%	2.10%	1.20%	65.80%	27.70%	9.40%	4.80%	3.70%
		15FF	55%	16.90%	3.70%	1.50%	0.70%	62.30%	27.10%	9%	4.20%	3.60%
	10	6 FF	60.90%	20.80%	6%	3.60%	2.20%	72.70%	9%	7.50%	6.10%	4.50%
		10 FF	60.10%	19.90%	5%	2.70%	1.50%	70.30%	30.80%	7.90%	5.20%	3.80%
		15FF	65.90%	22.30%	4.90%	2.10%	1.10%	66.20%	29%	7.50%	4.90%	3.70%
	2	6 FF	45.60%	8.40%	2.50%	1.60%	1.20%	59.50%	13.70%	5.50%	4.20%	3.30%
		10 FF	45.40%	9.20%	3%	1.80%	1.30%	64.40%	18.10%	7.80%	4.20%	3.10%
		15FF	46.20%	10.40%	3.70%	2.40%	1.70%	63%	17.20%	7%	3.60%	2.80%
	4	6 FF	51.40%	13.30%	3.60%	2.30%	1.60%	65.90%	19.80%	5.90%	4.20%	3.20%
SEMIFLEX		10 FF	50.30%	13%	3.10%	1.80%	1.30%	66%	22%	8.30%	4.70%	3.80%
		15FF	50.70%	13.40%	3.10%	1.60%	1.10%	64%	21.50%	7.40%	4.30%	3.20%
	8	6 FF	61.30%	22.10%	6%	3.90%	2.80%	72%	31.40%	8.60%	5.90%	4.50%
		10 FF	60%	21.10%	5.20%	3%	2.10%	69.60%	29.30%	7.80%	5.10%	4%
		15FF	59.90%	21%	4.90%	2.60%	1.80%	67%	23%	7.60%	4.60%	3%
	10	6 FF	65.60%	25.90%	7.20%	4.80%	3.40%	75%	36.30%	9.80%	6.80%	5.20%
		10 FF	64.80%	24.70%	6.20%	3.70%	2.50%	73.60%	34%	8.80%	5.80%	4.50%
		15FF	64.40%	24.60%	5.80%	3.20%	2.20%	69.90%	31%	8.60%	5%	4%

**Table 3:** Measured Relative Peripheral Dose for 6 MV FFF with Apex and Agility for the Field size 5x5 and 10×10 Using Pinpoint and Semiflex. (FFF: free flattening filter)

		Distance from the field edge									
Chamber	Depth (cm)	Apex					Agility				
		1cm	2cm	3cm	4cm	5cm	1cm	2cm	3cm	4cm	5cm
PINPOINT	2	28.60%	4.90%	1.70%	1.10%	0.80%	40.40%	12%	3.40%	2.20%	1.60%
10X10	4	35.10%	7.80%	2.30%	1.50%	1%	45.60%	12%	3.40%	2.20%	1.60%
	8	46.90%	13.60%	4.30%	2.80%	1.90%	54.70%	19.90%	5.50%	3.60%	2.70%
	10	52.50%	16.20%	5.20%	3.40%	2.40%	59.10%	23.60%	6.60%	4.40%	4.20%
	2	30.60%	3.60%	1.10%	0.60%	0.30%	49.50%	6.30%	1.60%	1%	0.70%
5X5	4	34.60%	6.00%	1.50%	0.90%	0.40%	50.70%	9.70%	2.20%	1.30%	0.40%
	8	45.20%	7.30%	2.50%	1.50%	0.80%	56.90%	16.50%	3.60%	2.10%	1.40%
	10	51.30%	7.60%	3.00%	1.90%	1.10%	58.00%	18.90%	4.20%	2.50%	1.70%
SEMIFLEX	2	36.40%	6.40%	2.10%	1.50%	1.10%	45.50%	8.90%	2.80%	2%	1.60%
10X10	4	42%	10.30%	2.30%	1.90%	1.40%	49.80%	13.60%	3.70%	2.50%	1.90%
	8	52%	17.30%	4.90%	3.30%	2.40%	54.10%	18.50%	4.90%	3.50%	2.70%
	10	56.70%	20.50%	5.90%	4.10%	3%	61.70%	26.20%	7.10%	4.80%	3.50%
	2	39%	4.70%	1.40%	1%	0.60%	53.70%	7.10%	1.80%	1.10%	0.80%
5X5	4	42.40%	12.70%	2.10%	1.10%	0.90%	57.10%	11.20%	2.40%	1.40%	1%
	8	47.50%	12.70%	3.10%	2%	1.30%	61%	18.20%	3.90%	2.30%	1.60%
	10	50.20%	15%	3.70%	2.40%	1.70%	62.90%	21.40%	4.60%	2.70%	1.90%

## Author Contribution Statement

Ms. Minnu N Tomy, Ms. Shambhavi C, Data collection and Article Drafting (Equal contribution). Dr Sarath s Nair, concept, design of the Work and critical revision, Sumeer Hussain, Dr Shirley Lewis article drafting, Dr Krishna Sharan, Jyothi, Dr Sajeesh S Nair critical revision of the article. .
